# Lipids in *Entamoeba histolytica*: Host-Dependence and Virulence Factors

**DOI:** 10.3389/fcimb.2020.00075

**Published:** 2020-03-10

**Authors:** Silvia Castellanos-Castro, Jeni Bolaños, Esther Orozco

**Affiliations:** ^1^College of Sciences and Humanities, Autonomous University of Mexico City, Mexico City, Mexico; ^2^BioImage Analysis Unit, Pasteur Institute, Paris, France; ^3^Department of Infectomics and Molecular Pathogenesis, Center for Research and Advanced Studies of the National Polytechnic Institute, Mexico City, Mexico; ^4^Centro Multidisciplinario de Estudios en Biotecnología, FMVZ, Universidad Michoacana de San Nnicolás Hidalgo, Morelia, Mexico

**Keywords:** *Entamoeba histolytica*, lipid metabolism, phospholipid synthesis pathway, cholesterol transport, virulence factors

## Abstract

Lipids are essential players in parasites pathogenesis. In particular, the highly phagocytic trophozoites of *Entamoeba histolytica*, the causative agent of amoebiasis, exhibit a dynamic membrane fusion and fission, in which lipids strongly participate; particularly during the overstated motility of the parasite to reach and attack the epithelia and ingest target cells. Synthesis and metabolism of lipids in this protozoan present remarkable difference with those performed by other eukaryotes. Here, we reviewed the current knowledge on lipids in *E. histolytica*. Trophozoites synthesize phosphatidylcholine and phosphatidylethanolamine by the Kennedy pathway; and sphingolipids, phosphatidylserine, and phosphatidylinositol, by processes similar to those used by other eukaryotes. However, trophozoites lack enzymes for cholesterol and fatty acids synthesis, which are scavenged from the host or culture medium by specific mechanisms. Cholesterol, a fundamental molecule for the expression of virulence, is transported from the medium into the trophozoites by EhNPC1 and EhNPC2 proteins. Inside cells, lipids are distributed by different pathways, including by the participation of the endosomal sorting complex required for transport (ESCRT), involved in vesicle fusion and fission. Cholesterol interacts with the phospholipid lysobisphosphatidic acid (LBPA) and EhADH, an ALIX family protein, also involved in phagocytosis. In this review, we summarize the known information on phospholipids synthesis and cholesterol transport as well as their metabolic pathways in *E. histolytica*; highlighting the mechanisms used by trophozoites to dispose lipids involved in the virulence processes.

## Introduction

Lipids have multiple roles in maintaining the structure and function of cellular membranes. Phospholipids provide flexibility, whereas sterols rigidity keeps membranes stability. Besides, lipids maintain membrane chemical composition. Lipids are also modulators of cellular adhesion points. This acquires special relevance in protozoan parasites that usually adhere to the target cell as one of the first steps during invasion (Das et al., 2002).

In the protozoan parasite *Entamoeba histolytica*, lipids are key molecules for virulence expression. *E. histolytica* is the causative agent of human amoebiasis, an enteric infection that affects nearly 50 million people worldwide and causes more than 55,000 deaths annually (Shirley et al., 2018). Cysts are excreted with human stools, contaminating water and food. Inside the intestine, the cysts become trophozoites, which multiply and invade the epithelium (Sehgal et al., 1996). Trophozoites again are transformed into cysts, which are released into the environment with the feces. In addition, from the intestine, trophozoites can also reach blood circulation and infect other organs, mainly the liver, where they produce abscesses (Sehgal et al., 1996).

*E. histolytica* trophozoites lack typical cellular structures present in higher eukaryotes, such as mitochondria, Golgi apparatus and a well-differentiated endoplasmic reticulum (Ghosh et al., 1999). The parasite uses primitive organelles that through a dynamic and complex vesicle trafficking perform their corresponding functions (Bañuelos et al., 2012), which remarks the importance of lipids in virulence. In addition, lipids also work as signaling molecules, energy stores, post-translational modifiers and inclusive, as virulence factors (Shrimal et al., 2012). Remarkably, they participate in membrane remodeling and synthesis, basic events for endocytosis and phagocytosis pathways, constitutive functions in trophozoites for nutrition, defense, and attack. Furthermore, lipids interact with proteins that act as adhesins, proteases, receptors, vesicle trafficking -promoting particles, and molecules involved in fission and fusion of membranes, among others (Das et al., 2002).

Pathogenesis of *E. histolytica* is characterized by sequential steps as mucus layer degradation, adherence to the epithelium, cytotoxic, and cytolytic events, phagocytosis, migration, and tissue invasion (Espinosa-Cantellano and Martinez-Palomo, 2000). All these processes are performed with the active participation of lipids (Espinosa-Cantellano and Martinez-Palomo, 2000; Byekova et al., 2010; Das and Nozaki, 2018). Trophozoites degrade the mucus layer by the secretion of glycosidases and cysteine proteases (Espinosa-Cantellano and Martinez-Palomo, 2000; Cuellar et al., 2017). Thanks its lipid composition, amebic membranes are resistant to the lytic compounds of the parasite (Andrä et al., 2004). Then, trophozoites adhere to the epithelium, through surface membrane molecules such as the Gal/GalNac lectin and the EhCPADH complex. The Gal/GalNac lectin is involved in adhesion to target cells and signaling (Mann, 2002). Significantly, cholesterol and sphingolipids, present in lipid rafts, participate in the organization of the three subunits that form this lectin (Welter et al., 2011). Cholesterol enhances the trophozoites adherence to the host cells and extracellular matrix (Goldston et al., 2012).

The EhCPADH complex, formed by a cysteine protease (EhCP112) and an adhesin (EhADH), is also involved in adherence to and destruction of the epithelium (Cuellar et al., 2017; Hernández-Nava et al., 2017). Through the cysteine protease, this complex destroys the mucus layer; and by the adhesin, it enhances the trophozoites adherence to the epithelium. Once adhered to the cells, EhCPADH opens the cellular junctions, provoking the epithelium destruction (Cuellar et al., 2017; Hernández-Nava et al., 2017). EhADH binds to cholesterol and the phospholipid lysobisphosphatidic acid (LBPA) (Bolaños et al., 2016; Castellanos-Castro et al., 2016), but the participation of lipid rafts in this protein functions has not been studied yet

Because of the high motility of trophozoites and its enzymatic activities, they can cross the epithelium and invade tissues, reaching the blood circulation, and damaging other organs, mainly the liver (Sehgal et al., 1996). Motility is a necessary event for invasion and phagocytosis, and lipids play a key role in these events, forming part of the distinct membrane vesicles composition (Coudrier et al., 2004; Thibeaux et al., 2013).

The myosin and actin-rich cytoskeleton network reacts to chemotactic signals, including target cells (Meza et al., 2006), provoking, with the active participation of different lipids, a dynamic membrane remodeling (Arhets et al., 1995). Then, cells polarize and form pseudopodia, uroids (the posterior appendix of membrane accumulation that is formed when trophozoites moves), blebs and vesicles that contribute in tissue colonization and phagocytosis (Arhets et al., 1995). This happens in a similar way to the events detected during metastasis and extravasation of transformed cells (Orozco et al., 1994; Ralston and Petri, 2011). Pseudopodia are rich in actin, myosin IB, and signaling molecules such as the phosphatidylinositol-3,4,5 triphosphate [PI(3,4,5)P_3_] (Voigt et al., 1999; Byekova et al., 2010). The uroid concentrates myosin II and signaling molecules (Arhets et al., 1995). Each one of these structures could have different lipid composition, that have not sufficiently studied yet.

In eukaryotes, blebs and pseudopodia formation participate in cell adhesion, metastasis, cytokinesis, and developmental stages (Burton and Taylor, 1997; Maugis et al., 2010). These functions require the disposal of lipids and proteins in reserve of the cell, or they are actively synthesized to supply the cellular requirements for new membranes (López-Reyes et al., 2010; Bañuelos et al., 2012; Avalos-Padilla et al., 2018). There are few studies on these events in *E. histolytica*, but it is plausible to hypothesize that synthesis and metabolism of lipids are activated during invasion and phagocytosis.

The endomembrane system in eukaryotes is a dynamic network capable of present curvature, folding and invaginations. These events permit vesicle fission and fusion to other membranes (Shin et al., 2012), allowing cell compartmentalization and membrane remodeling. In *E. histolytica*, cholesterol and fatty acids, necessary for these events, are not synthesized by the trophozoites; they are taken from the environment into the cells (Sawyer et al., 1967; Singh et al., 1971; Serrano-Luna et al., 2010; Bolaños et al., 2016). Once inside the cell, exogenous lipids are hydrolyzed by lipases and the products used as building material to produce molecules for the constant membrane renewal (Aley et al., 1980; Das et al., 2002). The AmoebaDB contains 22 potential homologs of lipid-transfer proteins. These proteins can be classified in 15 START-domain-containing proteins that bind to sterols, phospholipids or ceramides; four candidates with ORD-domain which binds to sterols and phosphatidylinositol-4 phosphate, two proteins with Sec14-domain that is known to bind to phosphatidylcholine (PC) and phosphatidylinositides, and a single PRELI-domain-containing protein that transfer phosphatidic acid (PA). All these data suggest the presence of a well-organized machinery for lipid transport in *E. histolytica* (Das et al., 2002; Das and Nozaki, 2018). However, experimental data are needed to prove this.

In this review, we have analyzed the known information on lipid composition, phospholipid metabolic pathways and cholesterol transport in *E. histolytica*, using published literature and KEGG genome databases (https://www.genome.jp/kegg/). We also discuss the scarce literature on lipid alteration in patients during the host-parasite interaction. Research on lipid synthesis and metabolism pathways may be a promising field to develop effective vaccines and anti-parasitic drugs.

## Lipid Composition in *E. histolytica* Trophozoites

Membranes of *E. histolytica* trophozoite are mainly composed by phospholipids and cholesterol (60–70% of the total lipids; Das et al., 2002), exhibiting differences to lipid proportions of mammalian cells (Table 1; Aley et al., 1980; van Meer and de Kroon, 2011). One of the main variances is related to the amount of sphingomyelin. In amoebae, this lipid is present in traces in plasma and internal membranes, while in mammalian cells it constitutes 23 and 16%, respectively. In contrast, the ceramide quantity is remarkably higher in the plasma membrane of trophozoites (38%) than in mammalian cells (Aley et al., 1980; Cerbón and Flores, 1981; van Meer and de Kroon, 2011). In most eukaryotes, the cholesterol: phospholipid ratio is 0.3 in whole cells (Vance, 2015), whereas, in *E. histolytica* purified plasma membranes have a 0.87 ratio (Aley et al., 1980). Glycerophospholipids proportions also differ in the parasite membrane: phosphatidylcholine (PC) is more abundant (56%) than in mammalian cells (42%); while the opposite happens with phosphatidylinositol (PI), which is 7% in lipids of mammalian membranes, and in trophozoites it appears only in traces (Aley et al., 1980; Cerbón and Flores, 1981; van Meer and de Kroon, 2011; Vance, 2015).

**Table 1:** Relative abundance of eukaryotic and amoeba lipids.

	Eukaryotes^*^		E. histolytica^**^	
	**%**		**%**	
Lipid	PM	IM	PM	IM
PC	43	42	13	56
PE^a^	21	21	34	19
PI	7	6	Traces	3
PS	4	Traces	8	4
SM	23	16	Traces	Traces
CAEP	Traces	Traces	38	18
Unidentified	nr	nr	9	Traces
**Ratio cholesterol:phospholipid**				
Cholesterol^b^	0.75	nr	0.87	nr

*PM, plasma membrane; IM, internal membranes*.

a *Sum of PE1 and PE2*.

b *Ratio cholesterol/phospholipid. Values are the percent of total lipid phosphorous*.

* *van Meer and de Kroon (2011)*,

** *Aley et al. (1980). nr, Not reported*.

There are only two reports about phospholipid proportions in *E. histolytica* trophozoites (Aley et al., 1980; Cerbón and Flores, 1981), which are summarized in Table 2. The ethanolamine phosphate (CEP), a ceramide, has been identified in trophozoites, and it represents the major part of total sphingolipids (SL) (65–75%) in amoebae (Cerbón and Flores, 1981). Other eukaryotes have lower quantities of ceramides; for example, *Drosophila melanogaster* has 15% and HeLa and CHO-KI cells only 0.03% (Cerbón and Flores, 1981; Vacaru et al., 2009). Phosphatidic acid (PA), the simplest phospholipid found as the precursor of glycerophospholipids and triacylglycerols (Vance, 1998), was identified in *E. histolytica* by standard migration in TLC experiments; however, its proportion and specific function are unknown (Aley et al., 1980). Interestingly, Aley et al. (1980) detected two unidentified spots in total lipids that were marked on the thin chromatography plate as “X” and “Y”. Together, they represent 9% of lipids of whole cells (Aley et al., 1980). Recently, by TLC and monoclonal antibodies, we detected the phospholipid LBPA in trophozoites (Castellanos-Castro et al., 2016), suspecting that it corresponds to the “X” spot, described by Aley et al. By confocal microscopy, LBPA was located in acidic vesicles co-localizing with cholesterol and EhADH and EhRab7proteins, both involved in phagocytosis (Nakada-Tsukui et al., 2005; Castellanos-Castro et al., 2016). The proportion of LBPA in whole cells has not been elucidated yet.

**Table 2:** Lipids in *Entamoeba histolytica*.

**Lipid**	**Abundance^**a**^**	**Abundance^**b**^**	**Function**	**References**
PE	14	19	Donor of polar heads to glycoconjugate lipoproteins. Vesicles fusion and fission	Wong-Baeza et al., 2010 Vance 2015
PS	9	8	Signaling Binding ESCRT to form ILVs It is associated with EhCaBP3 protein during phagocytosis	Avalos-Padilla et al., 2018 Aslam et al., 2012
PC	27	48	Membrane composition. Precursor of lytic molecules	Weltzien, 1979
PA	nr	nr	Binding phosphatidylcholine transfer protein-like (EhPCTP-l)	Piña-Vázquez et al., 2014
LBPA	nr	nr	Acidic vesicular trafficking	Castellanos-Castro et al., 2016
PI	7	5	Binding ESCRT Precursor of phosphoinositides	Avalos-Padilla et al., 2018 Sharma et al., 2019
PI(3,4,5)P_3_	nr	nr	Phagocytosis Locomotion	Ghosh et al., 1999 Meza, 2000
PI(4,5)P_2_	nr	nr	Pinocytosis and adhesion Present in lipid rafts, may regulate trophozoite motility Binds to cytoskeleton and maintain the cell shape and integrity	Ghosh et al., 1999 Koushik et al., 2013 Sharma et al., 2019
PI3P	nr	nr	Phagosomal cup formation	Nakada-Tsukui et al., 2009 Powell et al., 2006
SL	41	17	Lipid rafts formation Cellular adherence and fluid endocytosis	Goldston et al., 2012
SM	3	traces	Forming ceramides in parasite membranes	Mfotie Njoya et al., 2014
CAEP	10	16	Membrane stability	Cerbón and Flores, 1981
CEP	28	nr	Cytotoxicity protection	Cerbón and Flores, 1981
Unidentified	nr	9	nr	Aley et al., 1980
Cholesterol	nr	46^*^	Membrane composition Virulence properties	Goldston et al., 2012

*Relative abundance is given as % total lipid phosphorous*.

a *Cerbón and Flores (1981)*.

b *Aley et al. (1980)*.

* *Molar ratio cholesterol/mol of phospholipid, nr, Not reported*.

Some lipid functions studied in amoebae are similar to the ones described for other eukaryotes, but others have particular characteristics in *E. histolytica* (Laughlin et al., 2004; Powell et al., 2006; Byekova et al., 2010; Coskun and Simons, 2011). In Table 2, we show some of them. In summary, the particular lipid composition and proportions in different cellular compartments in trophozoites might be at the base of the great movement, the voracious phagocytosis and the virulence that this parasite could present, hence the importance of their study.

## Properties and Function of Phospholipids in *E. histolytica*

Within the fluid mosaic, phospholipids modulate water content, ion permeability and electrical capacitance of membranes, affecting structure and function of membranes (Singer and Nicolson, 1972). Their proportions differ in membranes according to the function that they perform (Vance, 2015). In *E. histolytica* trophozoites, phospholipids are crucial for vital functions, because the parasite organelles are distinct from the typical ones in other eukaryotes (Wiśniewska et al., 2003; Fagone and Jackowski, 2009; Avalos-Padilla et al., 2018) and most of its cellular functions are solved through a dynamic vesicular traffic.

### Glycerophospholipids

Phospholipids are classified in glycerophospholipids and sphingolipids (Sud et al., 2007). Glycerophospholipids have one glycerol backbone with two fatty acids (saturated or unsaturated) esterified to hydroxyl groups, and one phosphoryl group joined by a phosphodiester bond to a polar group which could be choline, inositol, ethanolamine, serine, or glycerol (Sud et al., 2007). Their synthesis pathway requires all these components, as well as the enzymes to assemble them (Cogan, 1966). In eukaryotes, including *E. histolytica* trophozoites, free fatty acids and glycerol groups are located mainly in the cytoplasm, and protein carriers are required to transport the polar heads during the phospholipid metabolism (Kent et al., 1991). *E. histolytica* has a simple machinery to perform some of the glycerophospholipid metabolic pathways (Jeelani and Nozaki, 2014).

#### Phosphatidylcholine (PC) and Phosphatidylethanolamine (PE)

##### PC and PE synthesis and metabolism

In *E. histolytica*, the most abundant glycerophospholipids are PC and PE (Aley et al., 1980; Cerbón and Flores, 1981). PC participates in the maintenance of the structural integrity and confers fluidity to membranes (van Meer and de Kroon, 2011). After hydrolysis, PC is the principal source of PA, lysophosphatidic acid (LPA), and signaling molecules, such as the second messenger diacylglycerol (DAG) and the arachidonic acid derivatives (Li and Vance, 2008; Gibellini and Smith, 2010).

PC and PE share the same synthesis pathways, since the same enzymes are involved in the synthesis of both phospholipids (Gibellini and Smith, 2010). Mammalian cells are not able to synthesize the choline and ethanolamine polar heads (Henneberry et al., 2002). These metabolites are taken from the medium by specific transporters such as hemicholinium-3-sensitive choline transporter (CHT), then, they are incorporated to the major route of phospholipid synthesis process known as Kennedy pathway, to be integrated into phospholipids PC and PE of cellular membranes (Gibellini and Smith, 2010). The source of choline and ethanolamine moieties in *E. histolytica* has not been studied. During PC and PE biosynthesis, choline and ethanolamine are phosphorylated by choline/ethanolamine kinases and then, activated by choline phosphate cytidyl-transferase and ethanolamine phosphate cytidyl-transferase. After this, CDP-choline/CDP-ethanolamine is transferred to diacylglycerol by choline/ethanolamine phosphotransferase to produce PC and PE (Gibellini and Smith, 2010). PE can be also methylated by phosphoethanolamine N-methyltransferase to produce PC (Henneberry et al., 2002). This mechanism is ubiquitously present in eukaryotes, including in some protozoa such as *Plasmodium, Trypanosoma, Leishmania*, and *E. histolytica* (Zufferey et al., 2002; Husain et al., 2010; Farine et al., 2015). *Giardia lamblia*, lacks the enzymes involved in Kennedy pathway (Gibellini and Smith, 2010).

Kennedy pathway was experimentally identified in *E. histolytica* and it is up regulated during L-cysteine deprivation (Husain et al., 2010; Jeelani and Nozaki, 2014). Under this culture conditions, PC and PE concentration increases in the cells, and, at the same time, trophozoites synthesize the unconventional phosphatidyl isopropanolamine phospholipid (PtdIspn) from methylglyoxal via the aminoacetone (Husain et al., 2010). PtdIspn is not detectable when trophozoites are cultured in 8 mM of L-cysteine, used in standard culture medium (Husain et al., 2010). The phospholipid alteration is reflected in changes in membrane integrity and fluidity, which may affect protein translocation across the plasma membrane (Husain et al., 2010). Enzymes involved in Kennedy pathway are reported in the *E. histolytica* genome in KEGG database: ethanolamine kinase (EHI_148580), ethanolamine phosphate-cytidyl-transferase (EHI_095120) and ethanolamine phosphotransferase (EHI_045570) (Table 3).

**Table 3:** Glycerophospholipids metabolism.

**Name**	**Entry**	**EC**
Lecithin-cholesterol acyltransferase	EHI_031360 **EHI_136400**	2.3.1.43
Lysophospholipid acyltransferase	EHI_086180	2.3.1.51
Lysophospholipase III	EHI_020250	3.1.1.5
Glycerophosphoryl diester phosphodiesterase	EHI_059880 **EHI_113970** **EHI_068320** **EHI_199860**	3.1.4.46
Phosphatidylethanolamine/phosphatidyl-N-methylethanolamine N-methyltransferase	EHI_153710	2.1.1.71
Diacylglycerol diphosphate phosphatase/phosphatidate phosphatase	EHI_165320	3.1.3.4
Diacylglycerol acyltransferase	EHI_099180	2.3.1.158
Phospholipase D1/2	EHI_082560 **EHI_146550** **EHI_146400**	3.1.4.4
Diacylglycerol kinase (ATP)	EHI_045610 **EHI_153440**	2.7.1.107
Phosphatidate cytidylyltransferase	EHI_054750	2.7.7.41
CDP-diacylglycerol-inositol 3-phosphatidyltransferase	EHI_069630	2.7.8.11
1-acyl-glycerol-3-phosphate acyltransferase	EHI_155730 **EHI_097610**	na
Cardiolipin synthase (CMP-forming)	EHI_035400	2.7.8.41
Phosphatidylserine synthase 2	EHI_009800	2.7.8.29
Ethanolaminephosphotransferase	EHI_045570 **EHI_182040** **EHI_067920**	2.7.8.1
Ethanolamine-phosphate cytidylyltransferase	EHI_095120 **EHI_140590**	2.7.7.14
Ethanolamine kinase	EHI_148580 **EHI_152340**	2.7.1.82

*In bold, paralogs identified in E. histolytica genome by Blast comparative analysis*.

*na, not assigned*.

In addition, the hypothetical genes for the alternative routes involving sphingolipids (SL) metabolism are also present in the *E. histolytica* genome. Sphingolipids metabolism is related to the PC and PE synthesis. First, sphingomyelin (SM) is catabolized by sphingomyelinases (SMA) to produce phosphocholine, which follows the Kennedy pathway (Gulati et al., 2015). In our search, we found a sphingomyelin phosphodiesterase in the *E. histolytica* genome (EHI_007460) and the previously reported acid sphingomyelinase-like phosphodiesterase (EHI_040600) (Table 4), detected by transcriptomic assays (Mfotie Njoya et al., 2014). On the other hand, several studies have shown that in eukaryotes SL metabolism is also the main source of ethanolamine, which is incorporated into Kennedy pathway to synthesize PE (Zufferey et al., 2002; Zhang et al., 2007). PE can be transformed into PC via phosphatidylethanolamine N-methyltransferase, as it happens in *Leishmania* (Peacock et al., 2007; Zhang et al., 2007). Furthermore, a hypothetical phosphatidylethanolamine N-methyltransferase (EHI_153710) is present in the *E. histolytica* genome (Table 3). All these findings suggest that trophozoites may supply their PE needs via Kennedy pathway and SL metabolism, and then, PE may be transformed to PC by phosphatidylethanolamine N-methyltransferase. The participation of all these mechanisms for membrane remodeling and surviving by trophozoites remains elusive.

**Table 4:** Sphingolipid metabolism.

**Name**	**Entry**	**EC**
Sphinganine-1-phosphate aldolase	EHI_039350	4.1.2.27
Sphingosine kinase	EHI_023410	2.7.1.91
Serine palmitoyltransferase	EHI_069310 **EHI_152280**	2.3.1.50
Sphingomyelin phosphodiesterase	EHI_007460 **EHI_088080** **EHI_193190** **EHI_067710** **EHI_022610** **EHI_103250**	3.1.4.12
Longevity-assurance family protein	EHI_139080^*^ **EHI_193400** **EHI_187230** **EHI_151290**	2.3.1.24
Longevity-assurance family protein	EHI_130860^*^	2.3.1.24
Acid sphingomyelinase-like phosphodiesterase	EHI_040600^*^ **EHI_100080** **EHI_118110** **EHI_125660**	3.1.4.12

*In bold, paralogs identified in E. histolytica genome by Blast comparative analysis*.

* *Identified by transcriptomic assays (Mfotie Njoya et al., 2014)*.

##### PC and PE in virulence

During the infection by *E. histolytica*, PC is deacylated to form lysophosphatidylcholine, which is a potent lytic molecule when trophozoites are in contact with target cells, particularly with the erythrocyte surface (Weltzien, 1979). On the other hand, PE is crucial for embedding membrane proteins and it is related to vesicle fusion and fission processes (Vance, 2015). PE is the principal donor of ethanolamine moieties in posttranslational modifications of macromolecules such as lipopolysaccharides (LPS) of bacteria plasma membrane (Raetz et al., 2007). Intriguingly, *E. histolytica* has a molecular homologous of bacterial LPS, known as lipophosphopeptideglycan (LPPG) (Wong-Baeza et al., 2010). This molecule is abundant in plasma membrane and has been directly related to the trophozoite virulence (Moody et al., 1998). In eukaryotes, ethanolamine is also involved in glycosylphosphatidylinositol (GPI) anchor, which allows the attachment of proteins to the cell surface (Cogan, 1966; Signorell et al., 2008). In *E. histolytica*, the Gal/GalNac lectin light subunit is a GPI anchor protein and it participates in the host-parasite interaction (Petri et al., 1987).

#### Phosphatidylserine (PS)

##### PS synthesis and metabolism

In eukaryotes, PS is a negatively charged phospholipid found in early endosomes and in the cytosolic side of the plasma membrane (van Meer et al., 2008). When cells undergo into apoptosis, it is exposed to the external face (Fadok et al., 1998). The anionic property of this phospholipid allows the interaction with proteins related to membrane deformation, vesicle formation, and signaling processes (Sun and Drubin, 2012). PS is synthesized in a single reversible reaction, consisting in the exchange of a polar head with PC or PE (Vance, 1998). This reaction is catalyzed by PS synthase 1 and 2, respectively. PS synthase 2 is present in *E. histolytica* genome (EHI_009800) (Table 3), suggesting that trophozoites synthesize PS from PE.

##### PS in virulence

During multivesicular bodies (MVBs) formation in *E. histolytica*, vacuolar protein sorting proteins (EhVPSs) of the endosomal sorting complex required for transport (ESCRT) machinery, exhibit higher affinity to PS than to other anionic phospholipids to form the intraluminal vesicles (ILVs) (Avalos-Padilla et al., 2018). Furthermore, PS appears to be involved in the first steps of the phagocytosis process in association with EhCaBP3 protein (Aslam et al., 2012).

### Phosphatidylinositol (PI) and Phosphoinositides

#### PI and Phosphoinositides Synthesis and Metabolism

In mammalian cells, PI is a minor component of the cytosolic side of cell membranes (Wengelnik et al., 2018). The inositol ring of this phospholipid can be phosphorylated once, twice, or three times in the hydroxyl groups to form seven different species: phosphatidylinositol monophosphate (PI-3P, PI-4P, and PI-5P), phosphatidylinositol bi-phosphate [PI(4,5)P_2_, PI(3,5)P_2_, PI(3,4)P_2_] and phosphatidylinositol tri-phosphate PI(3,4,5)P_3_ (Balla, 2013). All these products are known as phosphoinositides and can be reversibly modified by kinases and phosphatases, changing their cellular functions, which are related to signaling and membrane traffic (Balla, 2013). Furthermore, they accumulate in certain subcellular compartments forming lipid marks for membrane identities (Balla, 2013). Although PIs have been identified in *E. histolytica*, enzymes belonging to phosphatidylinositol phosphate kinase (PIPK) family have been poorly studied. *In silico* analysis revealed that *E. histolytica* lacks type- II PIPK homologous, but the parasite presents type I and III PIPK. PIPK type I produces PI(3,4,5)P_3_ from PI(4,5)P_2_. PI(4)P is produced from IP by the action of PI(4)K type III and also, PI can be phosphorylated to generate PI(3)P by class III PI3K (Sharma et al., 2019).

Important advances in the lipid knowledge of *E. histolytica* constitute the findings focused on the inositol group. These molecules could be synthesized by the parasites or up taken from the host environment. In *E. histolytica* the *ino1* gene encodes for L-myo-inositol 1-phosphate synthase (1-1-P synthase) (Lohia et al., 1999). This synthase produces L-myo-inositol 1-phosphate by the transformation of D-glucose-6-phosphate (Lohia et al., 1999). During *de novo* PI synthesis in eukaryotes, myo-inositol-phosphate is dephosphorylated by inositol 3-phosphate monophosphatase and transformed into myo-inositol, which is converted in PI with CDP-diacylglycerol in a reaction catalyzed by PI synthase (also called CDP-diacylglycerol-inositol 3-phosphatidyltransferase; Nuwayhid et al., 2006). In the *E. histolytica* genome, there is one CDP-diacylglycerol-inositol 3-phosphatidyltransferase (EHI_069630), an enzyme related to the synthesis of CDP-diacylglycerol, suggesting that PI synthesis pathway is conserved in this parasite. However, experimental evidence is necessary to confirm this.

#### PI and Phosphoinositides in Virulence

The inositol group forms part of PI, Gal/GalNac lectin, and lipophosphoglycanes involved in signaling, adhesion, virulence, and host immune response activation (Moody et al., 1998; Mann, 2002). The Gal/GalNac lectin is a heterotrimeric protein (Petri et al., 2002) that comprises three units: *hgl* (heavy subunit), *igl* (intermediate subunit) and *lgl* (light subunit), this latter is glycophosphatidylinositol anchored to the membrane (Mann, 2002).

In addition to the molecules above described as involved in adherence, there are two principal lipophosphoglycanes in *E. histolytica*: LPG and LPPG, both have phosphate saccharides and LPPG possesses a significant amino acid content (Moody et al., 1998). LPG and LPPG are involved in trophozoites adherence to the host cell and in cytotoxicity (Stanley et al., 1992). Virulent strains of *E. histolytica* exhibit higher concentrations of LPPG than the non-virulent strains (Bhattacharya et al., 2000). This fact has led to several authors to propose that LPPG molecule is involved in the parasite virulence (Bhattacharya et al., 2000). Furthermore, trophozoites treated with monoclonal antibodies against LPPG reduce their capacity to produce intestinal inflammation, reinforcing this assumption (Zhang et al., 2002). LPPG is an activator of the immune response by Toll-like receptor 2 and 4, and also, it protects trophozoites from the host immune response and promotes their survival within the host (Maldonado-Bernal et al., 2005; Wong-Baeza et al., 2010). Furthermore, it is an immunogenic molecule, since mouse and human infected with amoebae develop anti-LPPG antibodies (Acosta-Altamirano et al., 1986).

The inositol group is also the polar head of phospholipids PI. It is an anionic molecule with affinity for amoebic ESCRT proteins to favor vesicle formation during endocytosis and other cellular functions (Avalos-Padilla et al., 2018). PI3P, PI(4,5)P_2_, and PI(3,4,5)P_3_ phosphoinositides are involved in the phagocytic cup formation in trophozoites, but not in the initial host-pathogen interaction, neither at intermediate and late phases of phagocytosis and nor during pinocytosis (Powell et al., 2006; Byekova et al., 2010). These findings support the hypothesis that the parasite has vesicles with distinct composition, according to their function that in most cases is unknown. PI3P was identified in the phagocytic cups using FYVE (Fab1p, YOTB, Vac1p, and EEA1) domains fused to the green fluorescent protein (Powell et al., 2006; Nakada-Tsukui et al., 2009), as a lipid biosensor that recognize PI3P enriched membranes (Lemmon, 2008). The FYVE finger domains are conserved in eukaryotes, including in *E. histolytica;* and it was originally observed in Fab1p, YOTB, Vac1p, and EEA1 (Early Endosomal Antigen 1) proteins, which gave the name to these domains.

In eukaryotes, phosphoinositides connect the cytoskeleton to the plasma membrane. In addition, they act as second messengers modulating the activity of several cytoskeleton proteins (Di Paolo and De Camilli, 2006; Balla et al., 2009). In *E. histolytica*, PI(4,5)P_2_ is formed by the action on EhPIPKI enzyme and it binds to the cytoskeleton to maintain the cell shape and integrity (Sharma et al., 2019). PI(4,5)P_2_ is present in lipid rafts, where may regulate parasite motility (Das and Nozaki, 2018). PI(3,4,5)P_3_ has not been detected in most of the unicellular organisms, but it has been identified in *E. histolytica* and *Plasmodium* (Tawk et al., 2010). In *E. histolytica*, it was visualized by immunofluorescence assays using lipid biosensors such as the recombinant PH lipid-binding domain coupled to glutathione S-transferase (GST) and green fluorescent protein (GFP) (Nakada-Tsukui et al., 2009; Byekova et al., 2010). It appeared accumulated in extending pseudopodia and in phagocytic cups during erythrophagocytosis (Byekova et al., 2010). PI(4,5)P_2_ and PI(3,4,5)P_3_ are hydrolyzed by phospholipase C (PLC) and the products obtained, including IP3, DAG, and Ca^2+^ function as signaling molecules, which interact with other effector proteins (Kortholt et al., 2007). When *E. histolytica* makes contact with fibronectin, calcium and PI(3,4,5)P_3_, PLC concentration increases, suggesting that PLC activation is the beginning of a signaling pathway for locomotion and dissemination of the trophozoites during invasion (Meza, 2000).

#### Phosphatidylglycerol (PG), LBPA, and Cardiolipin (CL)

##### PG, LBPA, and CL synthesis and metabolism

PA is the precursor of phosphatidylglycerol (PG), which possesses two glycerol moieties with one fatty acid esterified in carbon 1. PG could remain as PG or be transformed in LBPA or cardiolipin (CL). LBPA is an isomer of PG, with one fatty acid chain esterified to each carbon 2 (Mortuza et al., 2003; Vance, 2015).

LBPA is also known as bis monoacylglycerol phosphate (BMP). At least six LBPA species have been found in lipid extracts of trophozoites (Castellanos-Castro et al., 2016). LBPA synthesis pathway involves the PA precursor, which receives the phosphatidyl group to form glycerol-3-phosphate (G3P) by the phosphatidylglycerol phosphate (PGP) synthase action, also named CDP-diacylglycerol-glycerol 3P-3- phosphatidyl transferase (Hullin-Matsuda et al., 2007). Then, G3P is dephosphorylated to form PG by PGP phosphatase. PG could be transformed into its LBPA isomer by the action of transacylases and phospholipases (Hullin-Matsuda et al., 2007; Vance, 2015). The synthesis pathway of LBPA in *E. histolytica* is unknown. We have not found in the genome database the enzymes involved in the conversion of PGP to PG. However, the amoebic enzymes lysophospholipid acyltransferases (EHI_086180) and a putative 1-acyl-glycerol-3-phosphate acyltransferase (EHI_155730) are located in the KEGG database. As in other cases, experimental results will elucidate whether these enzymes are involved in LBPA synthesis in the parasite, as it happens in mammals. On the other hand, in other eukaryotes, PG can also receive an additional phosphatidyl group to form the cardiolipin (CL) by the CL synthases action (Gohil et al., 2005). PG and CL are enriched in bacteria and also are specific phospholipids of mitochondria (Hiraoka et al., 1993). They participate in the maintenance of protein structure and function in eukaryotes (Bligny and Douce, 1980). In prokaryotes, CL is the 5% and PG the 20–25% of total phospholipids (Hiraoka et al., 1993). *Giardia* trophozoites also have PG, forming 10.4% of the total phospholipids, although this parasite lacks mitochondria (Ellis et al., 1996). It is possible that in protozoa, the metabolism of this phospholipid differs from other eukaryotes. To date, PG and CL have not been found in *E. histolytica*. Intriguingly, trophozoites genome predicts one gene encoding for cardiolipin synthase CMP-forming (EHI_035400) (KEGG). A recent publication reported that in *Escherichia coli*, CL can be produced from PE by a cardiolipin synthase (ClsC) (Jeucken et al., 2018), which have a phospholipase D activity and hydrolyze many phospholipid types to produce PA and alcohols. Because a putative gene encoding for this enzyme is present in the *E. histolytica* genome (EHI_082560), it is logical to assume that CL could be synthesized by this way. However, experiments to define this are necessary as well to perform new strategies to investigate whether other molecules in the parasite carry out CL and PG functions in amoebae.

##### LBPA in virulence

*E. histolytica* LBPA was detected in endosomes during dextran uptake and erythrophagocytosis, associated with EhRab7A and EhADH proteins, suggesting that these lipids have similar functions to the ones described in mammalian cells (Nakada-Tsukui et al., 2005; Bissig and Gruenberg, 2013; Castellanos-Castro et al., 2016).

### Recycling of Glycerophospholipids in *E. histolytica*

Eukaryotic organisms have developed energy saving mechanisms such as the recycling of certain lipids and membrane compounds. Phospholipids can be hydrolyzed by phospholipases at a specific position of ester linkages producing free fatty acids and lysophospholipids (LPL), which can be re-used by other enzymes to synthesize new molecules or remodel phospholipids by reacylation reactions (Das et al., 2002; Vance, 2015). The deacyl/reacylation process is called Land's cycle and it is used by most of the cells to produce new or altered patterns of phospholipids and fatty acids, protecting cells from the accumulation of potentially toxic LPL and fatty acids (Yamashita et al., 1997; Das et al., 2001). During membrane remodeling, lysophospholipids have a short life. When its concentration is altered; the cellular membrane loses its properties and could be lysed (Yamashita et al., 1997). Land's cycle consists of two steps: deacylation of phospholipids by phospholipases and reacylation of lysophospholipids by acyltransferases (AT) and transacylases (TA) enzymes (Yamashita et al., 1997).

Phospholipases are present in hydrophobic regions and they could be soluble or membrane-associated proteins (Dennis, 1994). AT change the position or substitute saturated fatty acids at the sn1 position and unsaturated fatty acids at the sn2 position, altering the patterns of existing glycerophospholipids (Yamashita et al., 1997). Transacylation reactions performed by TA enzymes, consist in the transfer of fatty acids from glycerophospholipids to lysophospholipids (Yamashita et al., 1997). These reactions can be CoA-dependent or -independent in higher eukaryotes (Yamashita et al., 1997).

In *E. histolytica*, phospholipase A1 is a calcium-independent enzyme, with an optimal activity at pH 4.0. In contrast, phospholipase A2 is involved in regulation of cell activities with calcium requirements (Long-Krug et al., 1985). *E. histolytica* homogenates present phospholipase A1 activity in soluble proteins and plasma membrane fractions (Long-Krug et al., 1985). Whereas, calcium-dependent phospholipase A2 is present in soluble, plasma membrane and non-vesicular membrane fractions (Vargas-Villarreal et al., 1995). Thus, both phospholipases have their activity at the cell surface. This activity has been confirmed by the use of specific inhibitors (Vargas-Villarreal et al., 1995). The presence of AT and TA enzymes in *E. histolytica* have not been proved. However, in the parasite genome, there are hypothetical enzymes related to AT, TA, and other phospholipases such as: lysophospholipid acyltransferase (EHI_086180), lysophospholipase III (EHI_020250), glycerophosphoryl diester phosphodiesterase (EHI_059880), phospholipase D1/2 (EHI_082560), phospholipid: diacylglycerol acyltransferase (EHI_099180) (Table 3).

#### Recycling of Glycerophospholipids in Host-Parasite Interactions

*Giardia* is not able to *de novo* synthesize phospholipids, fatty acids or sterols. However, this parasite recycles the lipids that were up taken by the Land's cycle to get its phospholipid requirements (Das et al., 2001). Although the detailed mechanisms of the Land's cycle in the *E. histolytica* are unknown, some publications suggest their active participation in the lipid metabolism and pathogenesis processes (Das et al., 2002; Castellanos-Castro et al., 2016).

One idea that supports the presence of the Land's cycle in *E. histolytica* arises with the identification of the LBPA phospholipid, which is formed when PG is altered by the action of PL and TA (Vargas-Villarreal et al., 1995; Amidon et al., 1996; Castellanos-Castro et al., 2016). During early steps of phagocytosis and pinocytosis, LBPA decreases in trophozoites vesicles, suggesting that it is used as a donor of fatty acids to build other molecules (Castellanos-Castro et al., 2016). In addition, trophozoites are able to synthesize prostaglandin E2 (PGE2), which implies the deacylation/reacylation cycle for the arachidonic acid metabolism (Das et al., 2002). PGE2 participates in the parasite growth and tissue penetration, as well as in the evasion of the host immune response (Das et al., 2002). Prostaglandin and eicosanoid metabolism have been also identified in *Trypanosoma brucei* and *Schistosoma mansoni*. All these data support the important role that Land's cycle plays in host-parasite interactions (Nevhutalu et al., 1993; Eintracht et al., 1998).

**Figure 1:**
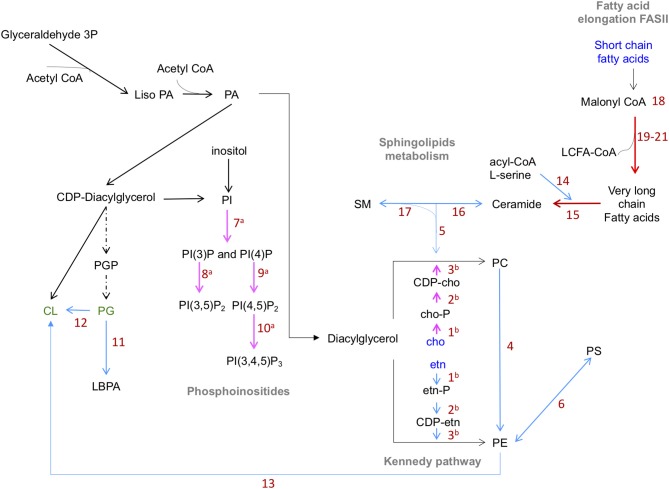
Predicted lipid synthesis pathways of phospholipids in *E. histolytica*. Blue lines: steps catalyzed by enzymes reported in KEGG database. Red lines: enzymes found by proteomics or transcriptomics analyses with inferred function. Pink lines: enzymes previously reported. Dotted black lines: enzymes not found in genome database. Abbreviations in blue: compounds up taken from the medium. Abbreviations in green: phospholipids not yet identified. PE biosynthesis: ethanolamine is phosphorylated by ethanolamine kinases (1) and activated by ethanolamine phosphate cytidyl-transferase (2). CDP-ethanolamine is transferred to diacylglycerol by ethanolamine phosphotransferase (3) to produce PC and PE. PE, methylated by phosphoethanolamine N-methyltransferase (4), produce PC. SM, catabolized by sphingomyelinases (SMA) (5), produce phosphocholine, which follows the Kennedy pathway. PS is synthesized from PE by PS synthase 2 (6). PI(4)P and PI(3)P are produced from IP by the action of type III EhPI(4)K and EhPI3K respectively (7). PI(3,5)P_2_ is generated from PI(3)P by the action of EhPIPKIII (8). PI(4,5)P_2_ is produced from PI(4)P by EhPIPKI (9). EhPI3KC1 (10) produces PI(3,4,5)P_3_ from PI(4,5)P_2_. Enzymes involved in the production of PG were not detected in *E. histolytica* genome. PL, AT, and TA enzymes (11) produce LBPA from PG. Cardiolipin is produced from PG or PE by synthase CMP-forming (12) and phospholipase D respectively (13). In sphingolipid metabolism, the enzymes involved are SPT (14), Cer S and its orthologs LAG family proteins (15) are involved in the production of ceramides, which can be transformed in complex SL by SL synthase (16). ASM participate in the ceramide's synthesis by the hydrolysis SM (17). Enzymes involved in fatty acid synthesis pathway were not detected in amoeba genome. Four putative enzymes that participate in the fatty acid elongation cycle: 3-ketoacyl-CoA (18), very-long-chain 3-oxoacyl CoA reductase (19), very-long-chain-3-hydroxyl-CoA dehydratase (20), and very-long-chain enoyl-CoA reductase (21). AcetylCoA, Acetyl coenzyme A; PA, Phosphatidic acid; etn, ethanolamine; CDP-, Cytidine diphosphate; cho, choline; -P, phosphorous; PC, phosphatidylcholine; PE, phosphatidylethanolamine; PS, phosphatidylserine; PG, phosphatidylglycerol; PI, phosphatidylinositol; (PI(4,5)P_2_, PI(3,5)P_2_), phosphatidylinositol bi-phosphate; (PI(3,4,5)P_3_), phosphatidylinositol (3,4,5) tri-phosphate; CL, cardiolipin; LBPA, lysobisphosphatidic acid; SM, sphingomyelin; PIPK, phosphatidylinositol phosphate kinases; AT, acyltransferases; TA, transacylases; PL, phospholipases; SPT, serine palmitoyl transferase; Cer S, ceramide synthase; LAG, longevity-assurance; ASM, acid sphingomyelinases (Husain et al., 2010; Sharma et al., 2019).

In summary, based on genome predictions, *E. histolytica* could synthesize most of the glycerophospholipids that the parasite requires (Figure 1). In addition, it is possible that the parasite also uses the molecular recycling mechanism to obtain material for the constitutive membrane remodeling occurring during many processes involved or not in the virulence mechanism of trophozoites.

### Sphingolipids (SL)

#### SL Synthesis and Metabolism

SL are ubiquitous lipids in eukaryotic cell membranes, mainly in plasma membrane (Merrill and Sandhoff, 2002). They participate in membranes structure, domain formation, signal transduction, cell growth, differentiation, cell polarization, cellular trafficking, and apoptosis (Mina and Denny, 2018). SL are enriched in lipid rafts, since they are amphipathic molecules that interact with sterols and other sphingolipids to form aggregates within membranes through intra and intermolecular hydrogen bonds (Bruzik, 1988; Futerman and Hannun, 2004). This property allows the exchange of proteins associated to the membrane with lipid anchors and specific protein-lipid interactions (Bruzik, 1988). The three components of SL structures are (i) a backbone consisting in a long-chain amino alcohol called sphingosine, (ii) one fatty acid (saturated or unsaturated) bound by an amide linkage, and (iii) a polar head group (Futerman and Hannun, 2004). There are many types of sphingolipids; the simplest are formed when the sphingosine backbone is N-acylated to form ceramides (Merrill and Sandhoff, 2002). Ceramides are the precursors for the complex SL synthesis. The most abundant in mammals is SM (Merrill and Sandhoff, 2002). As we mentioned above, SL metabolism shares some facts with glycerophospholipids biosynthesis (Holthuis and Menon, 2014). In mammals and some protozoa belonging to *Mastigophora, Kinetoplastids*, and *Apicomplexa*, the SL synthesis is performed by different enzymes (Ramakrishnan et al., 2013). Their biosynthesis process is simplified into three steps: (i) It starts in the ER with the condensation of acyl-CoA and L-serine by serine palmitoyl transferase (SPT), producing dihydrosphingosine (van Meer and Holthuis, 2000). (ii) Then, ceramide synthase (Cer S) acylates dihydrosphingosine, forming dihydroceramide, which is desaturated to ceramide (van Meer and Holthuis, 2000). (iii) Finally, ceramides are transported to Golgi to be transformed in complex SL by SL synthase (van Meer and Holthuis, 2000). Ceramides are different in mammals, plants, fungi and protozoa, thus, SL synthases are not the same in all eukaryotes (Merrill, 2011). In mammals, there is only one enzyme known as SM synthase (Merrill, 2011). Plants, fungi, and protozoa have other, called ceramide inositol phosphoryl ceramide (IPC) synthase (Holthuis and Menon, 2014; Mina and Denny, 2018). IPC has not been identified in *E. histolytica* and we did not find it in KEGG data base.

Acid sphingomyelinases (ASM) are enzymes that participate in the ceramides synthesis, by the hydrolysis of long chain SM (Kitatani et al., 2008). ASM are located within lysosomes and late endosomes during membrane turnover (Kitatani et al., 2008). ASM can be translocated from cellular compartments to the cell membrane where they synthesize ceramides from SM, reorganizing molecules and forming lipid-rafts that expose signaling proteins to induce cell death (Kitatani et al., 2008).

There is biochemical evidence of sphingolipids metabolism in amoebae, since they are capable to efficiently degrade and re-synthesize CEP (Cerbón and Flores, 1981). Furthermore, several genes encoding for hypothetical enzymes related to ceramides metabolism have been detected by transcriptomic analyses after incubation of trophozoites with extracts of *Codiaeum variegatum* extracts (Table 4; Mfotie Njoya et al., 2014). This plant, endemic of Cameroon, have been used as an effective treatment against bloody-diarrhea (Moundipa et al., 2005). In these studies, trophozoites showed RNA overexpression of two genes encoding for longevity-assurance (LAG) family proteins (EHI_139080 and EHI_130860), orthologs of ceramide synthase. In the same experiments, authors detected that an acid sphingomyelinase-like phosphodiesterase (EHI_040600) was down regulated. They also found several hypothetical long chain fatty acid synthases (Mfotie Njoya et al., 2014). Furthermore, the *E. histolytica* genome presents SPT (EHI_069310) and sphingosine kinase (EHI_023410), enzymes involved in sphingolipids metabolism (Table 4).

All these data support that SM metabolism has a role in forming the huge amount of ceramides in the amoebae membranes (Mfotie Njoya et al., 2014). Although these enzymes have not been experimentally explored, theses authors postulated that there is a high enzymatic activity of ceramide biosynthesis during the life cycle of trophozoites (Mfotie Njoya et al., 2014), as it happens in Trypansoma, Leishmania and Plasmodium (http://www.llamp.net/). Mfotie Njoya et al. (2014) also suggested that alteration in genes expression observed in trophozoites treated with C. variegatum extracts, produces accumulation of ceramides in the plasma membrane. These events cause lipid-rafts disorganization responsible of cell death and cell growth inhibition. Reduction of fatty acid metabolism and lipid trafficking is also altered in *E. histolytica* trophozoites treated with this plant (Mfotie Njoya et al., 2014).

#### SL in Virulence

Lipid rafts in *E. histolytica* are associated to some virulence processes. These SL-enriched membrane microdomains facilitate attachment of trophozoites to host tissues and regulate fluid phase endocytosis (Goldston et al., 2012).

Trophozoites of *E. histolytica* have a high ceramide proportion in comparison to mammalian cells. The high proportion of the CAEP in plasma membrane is unusual in higher eukaryotes (Cerbón and Flores, 1981). CAEP is resistant to hydrolases and is present in *E. invadens* and *E. dispar* (McLaughlin and Meerovitch, 1975). The high concentration of CAEP could be involved in plasticity and stability of trophozoite membranes, since they interact with many proteins through hydrogen bonds (Cerbón and Flores, 1981). CEP is also resistant to hydrolysis and protects trophozoites from their own proteases and the adverse conditions during the tissue invasion process (Cerbón and Flores, 1981).

## Fatty Acids Synthesis

Early studies reported that total lipids of *E. histolytica* trophozoites contain 50–60% of saturated fatty acids (Sawyer et al., 1967). In general, these molecules are formed by 12–24 carbons (Sawyer et al., 1967). The most abundant is the palmitic acid with 16 carbons (33.8%) (Sawyer et al., 1967). In eukaryotes, palmitic acid is produced during fatty acid synthesis and it is a precursor of longer molecules (Ramakrishnan et al., 2013). In amoebae, unsaturated fatty acids proportion is around 40–50% and they possess 16–24 carbons (Sawyer et al., 1967). The average of unsaturation in trophozoites fatty acids is between 1 and 4, including one odd fatty acid with 21 carbons and 1 unsaturation. This latter represents the 10.9% of total fatty acids (Sawyer et al., 1967). Experiments using trophozoites cultured in medium supplemented with isotopically labeled glucose, showed that 10% of the label appeared in lipid carbons and 77% in fatty acids fractions, suggesting that amoebae are not able to synthesize and extend fatty acid chains (Sawyer et al., 1967). Furthermore, in the same study, the authors found that *E. histolytica* fatty acids profiles differ from those reported in *Acanthamoeba* and *Hartmennella rhysodes* that can grow in medium without fatty acids (Sawyer et al., 1967). Besides, the *E. histolytica* genome has not the enzymes involved in fatty acid synthesis pathway. However, there are three putative enzymes (Table 5) that participate in the fatty acid elongation cycle (FAE) (Ramakrishnan et al., 2013): (i) the very-long-chain 3-oxoacyl CoA reductase (EHI_165070), (ii) the very-long-chain-3-hydroxyl-CoA dehydratase (EHI_110570) and (iii) the very-long-chain enoyl-CoA reductase (EHI_045030). Moreover, studies using proteomic approaches, reported the presence of two 3-ketoacyl-CoA synthases (EHI_158240 and EHI_112870) in purified trophozoite endomembranes (Perdomo et al., 2015), suggesting that *E. histolytica* is able to synthesize very long chain fatty acids from shorter fatty acids up taken from medium. In eukaryotes, FAE pathway is involved in the generation of very long chain fatty acids from malonyl-CoA, which is the source of carbon (Hiltunen et al., 2009). Ketoacyl-CoA synthases participate in the condensation of acyl CoA with malonyl-CoA to form 3-ketoacyl-CoA producing the acyl chain with two carbon atoms longer than before (Hiltunen et al., 2009). By LC/MS/MS, the presence of at least nine long-chain acyl-CoA synthase/ligase putative molecules were detected in *E. histolytica* membranes (Perdomo et al., 2015). In eukaryotes, these enzymes participate in the fatty acyl-CoA synthesis and in the fatty acid activation step before being incorporated into phospholipids and to start the beta-oxidation (Soupene and Kuypers, 2008). They play an important role in suppress fatty acid synthesis and are related to SL metabolism (Faergeman and Knudsen, 1997). To date, research on *E. histolytica* fatty acids metabolism and its connection with ceramide biosynthesis is still open to be explored.

**Table 5:** Enzymes involved in fatty acid elongation.

**Name**	**Entry**	**EC**
17 beta-estradiol 17-dehydrogenase/very-long- chain 3-oxoacyl-CoA reductase	EHI_165070	1.1.1.330
Very-long-chain (3R)-3-hydroxyacyl-CoA dehydratase	EHI_110570	4.2.1.134
Very-long-chain enoyl-CoA reductase	EHI_045030	1.3.1.93
Fatty acid elongase (3-ketoacyl-CoA synthase	EHI_158240^*^	na
Fatty acid elongase (3-ketoacyl-CoA synthase	EHI_112870^*^ **EHI_111000** **EHI_092190** **EHI_009370**	na

*In bold, paralogs identified in E. histolytica genome by Blast comparative analysis*.

* *Identified by proteomic approaches (Perdomo et al., 2015). na, not assigned*.

A crucial step in the understanding of lipid metabolism in *E. histolytica* is the recent identification of CoA synthesis pathway and the characterization of the pantothenate kinase (PanK), the first enzyme involved in this process (Nurkanto et al., 2018). In eukaryotes, CoA participates as acyl group carrier and carbonyl-activating group during fatty acid metabolism, the tricarboxylic acid cycle and other intermediary metabolic reactions. It is synthesized from pantothenate, cysteine and ATP (Leonardi and Jackowski, 2007). All the genes participating in CoA synthesis are expressed in *E. histolytica* trophozoites and *E. invadens* (Nurkanto et al., 2018). After silencing of *PanK* gene, a marked reduction of CoA concentrations and cellular growth retardation in trophozoites occurred (Nurkanto et al., 2018). Since most eukaryotes supply the pantothenate from the diet and PanK is not essential for cellular survival, this kinase could be a good chemotherapeutic target for anti-*E. histolytica* drugs (Nurkanto et al., 2018).

With all the information presented in this section, we assume that there are many open questions related to *E. histolytica* lipid metabolic pathways, which seem to be simpler than in other eukaryotes and the enzymes involved may differ from their orthologous described for other organisms (Figure 1).

## Cholesterol

### Cholesterol Synthesis and Metabolism

Cholesterol has a fundamental role in the organization, dynamics, and function of the cell membrane (Schroeder et al., 1990). It is essential for the maintenance of lipid rafts, acting as platforms to coordinate signal transduction events that allow the entry of pathogens to the host cells (Bansal et al., 2005). In mammalians, cholesterol can be obtained by two ways: (i) Enterocytes and hepatic cells can *de novo* synthetize this sterol in endoplasmic reticulum, (ii) however, most cells uptake cholesterol from low density lipoproteins (LDL) that are present in the serum. The LDL is internalized through the LDL receptor (RLDL) and then it passes throughout the endocytic compartments, where cholesterol esters are hydrolyzed by acid lipases allowing its separation from LDL (Ikonen, 2008). Endolysosome vesicles possess Niemann-Pick type C1 and C2 proteins (NPC1 and NPC2), which bind and transport cholesterol and SL to the cellular organelles (Davies, 2000). Alteration of these proteins causes the Niemann Pick diseases in humans.

*E. histolytica* needs cholesterol and phospholipids to carry out the biosynthesis of internal and plasma membranes and for other functions. However, the parasite is unable to synthesize cholesterol (Das et al., 2002). Trophozoites take this lipid from the milieu in cultured cells and from the intestine and liver cells of live organisms (Bansal et al., 2005; Serrano-Luna et al., 2010). According to the KEGG database, *Homo sapiens* has 25 enzymes for the sterols biosynthesis, *D. discoideum* 18, and *A. castellani* 21. In contrast, *E. histolytica* does not present any. This reinforces the fact that trophozoites take cholesterol from the environment to be incorporated and transported through different organelles, using EhNPC1 and EhNPC2 proteins, recently reported by us and discussed below (Bolaños et al., 2016).

#### EhNPC1, EhNPC2, and LPBA Networking: A Model for Cholesterol Capture, Internalization, and Distribution

Even when *E. histolytica* does not possess the machinery for cholesterol synthesis, it has mechanisms to internalize this sterol from the medium. Since 2012 it was described the presence of TMK39, a protein involved in cholesterol uptake. However, it is probably in association with other molecules, because TMK39 does not present cholesterol binding domains (Christy et al., 2012).

Recently, we identified the *Ehnpc1* and *Ehnpc2* genes, which encode for EhNPC1 and EhNPC2 proteins (Bolaños et al., 2016). *In silico* analyses, evidenced that EhNPC1 and EhNPC2 use the “Hand-off” model (Wang et al., 2010) to carry out cholesterol transferring. Serum pulse-chase experiments evidenced that EhNPCs proteins are concentrated in the cell periphery decorating the plasma membrane to capture and internalize the exogenous cholesterol (Bolaños et al., 2016). In steady state, unlike mammalian cells, where NPC1 and NPC2 are in late endosomes, EhNPC1 is localized mainly in the plasma membrane and EhNPC2 is in cytoplasmic vesicles. Both proteins are secreted and participate in internalization and cellular distribution of cholesterol (Bolaños et al., 2016). During phagocytosis, EhNPC2 sense cholesterol outside the trophozoite. At the same time the transmembrane EhNPC1 forms a channel for the entry of cholesterol bound to EhNPC2. Then, both proteins interact to share between them the cholesterol and transport it to MVBs. From MVBs, cholesterol is distributed to different cellular fates (Wang et al., 2010). Through confocal microscopy assays, digested erythrocytes appeared with cholesterol and EhNPCs proteins in huge vacuoles that correspond to phagolysosomes and MVBs (Bolaños et al., 2016). In addition, when endocytosis is in process, EhNPC2 interacts with EhSERCA in the endoplasmic reticulum. Both EhNPCs interact with EhRab7A, EhADH, and LBPA in late endosomes/lysosomes and in MVBs (Bolaños et al., 2016; Castellanos-Castro et al., 2016). These molecular interactions suggest that all they may be involved in lipid transport from MVBs to lysosomes or Golgi (Bolaños et al., 2016).

In mammalian, the transference of cholesterol between the NPCs proteins is favored by the acidic pH and the presence of LBPA in the late endosomes (Kobayashi et al., 1999; Ko et al., 2003). There, LBPA is in ILVs associated with Alix protein that regulates the cholesterol concentration in late endosomes (Chevallier et al., 2008; Scott et al., 2014). The role of Alix was demonstrated because silencing of the gene produces a reduction in the IVLs number and in the amount of LBPA and cholesterol. LBPA also participates in an alternative way to export cholesterol from late endosomes (Bissig and Gruenberg, 2014). Due to the fact that EhNPCs and LBPA were found associated in MVBs during phagocytosis of *E. histolytica*, we suggest that these molecules may present the same functions as mammalian cells.

### Cholesterol in Virulence

*E. histolytica* shows an enhanced virulence when is cultured with high cholesterol concentration (Serrano-Luna et al., 2010) This have been related with the lipid raft arrangements where an enrichment of the Gal/GalNAc lectin is present, producing a stronger adherence to target cells (Welter et al., 2011). Furthermore, it associates with the EhADH adhesin. All these molecules together with lipid rafts formation are crucial for vesicle formation, endocytosis and movement (Mittal et al., 2008). On other hand, cholesterol transport process by EhNPCs proteins impacts in motility and phagocytosis events, promoting the membrane synthesis and vacuoles fusion for virulence expression (Bolaños et al., 2016).

## Lipids Alteration in Patients Infected with *E. histolytica*

*E. histolytica* pathogenesis causes intestine damage associated with chronic inflammation, mucosal disruption, altered barrier integrity, and dysbiosis that compromise the correct nutrient absorption and provokes other pathologies: (i) become patients more susceptible to other chronic infections (Korpe and Petri, 2012), (ii) increase the possibilities to alter the microbiota and eukaryome in the intestine, (iii) 93% of patients with amoebic liver abscess (ALA) present changes in serum lipid profiles, mainly hypocholesterolemia, (iv) some studies have found abnormal cephalin-cholesterol flocculation test (Viranuvatti et al., 1963; Gujral et al., 1982; Flores et al., 2014; Filippas-Ntekouan et al., 2017) and significantly lower levels in the lipid profile in infected patients with ALA (Bansal et al., 2005), (v) triglycerides and cholesterol increase in the liver of hamsters infected with *E. histolytica* trophozoites, but animals show hyperlipidemia and hypocholesterolemia (Gujral et al., 1982). However, further research efforts need to be focused to better understanding the molecular mechanism on cholesterol alterations and lipid homeostasis changes during trophozoite infection. To date, it has not been proposed a mechanism on how *E. histolytica* could be involved in hypocholesterolemia, however, paradoxically, it is well-known that trophozoites grown in medium with high levels of cholesterol present an increased virulence (Olivos et al., 2005; Serrano-Luna et al., 2010). The cholesterol flux in mammalian host is throughout the entero-hepatic circulation, and, *E. histolytica* invades the liver by the same pathway, suggesting that the parasite colonizes cholesterol enriched organs, as described previously (Flores et al., 2014).

## Concluding Remarks

Lipids are essential in cellular processes and pathogenesis of *E. histolytica*. Lipid metabolic characterization in this parasite has been growing using complex technologies approaches: Genomes databases, gene silencing, transcriptomic, and proteomic analysis are giving important information about the enzymes and metabolites in parasite during infection. In this review, we took advantage of the available data to analyze the current knowledge of lipids in *E. histolytica*. This protozoan has a particular lipid composition. Its differences with mammals provide excellent targets for diagnosis and vaccine strategies that, up to date, have been poorly explored. *E. histolytica* has 27 putative enzymes involved in the lipid metabolic pathways, reported in KEGG database, the majority poorly studied. Although, in comparison with human, the number of enzymes found in *E. histolytica* seems to be low, trophozoites have mechanisms to synthesize or remodel phospholipids. They acquire cholesterol and fatty acids from the environment. Thus, trophozoites are host-or culture medium-dependent to get these lipids. Amoebic infection provokes alterations in host metabolism with impact in their serum lipid profiles, suggesting that parasites need lipid scavenging from host to regulate their own cellular processes for surviving, or may be the parasite and its molecules provoke disequilibrium in lipid synthesis and metabolism in the organism. However, lipid metabolism during host-pathogen interaction research is still an open field to be explored and exploited for the development of new methodologies for diagnosis and treatment.

## Author Contributions

All authors listed have made an extended and intellectual contribution to the work, revised, and approved it for publication. SC-C, JB, and EO: conception design, organized the database, and wrote sections.

### Conflict of Interest

The authors declare that the research was conducted in the absence of any commercial or financial relationships that could be construed as a potential conflict of interest.

## Abbreviations

ASM: Acid sphingomyelinase
AT: Acyltransferase
ALA: Amoebic liver abscess
BMP: Bis monoacylglycerol phosphate
CAEP: Ceramide aminoethyl phosphonate
CL: Cardiolipin
CEP: Ceramide ethanolamine phosphate
CAEP: Ceramide aminoethylphosphonate
Cer S: Ceramide synthase
CDP: Cytidine diphosphate
CoA: Coenzime A
DAG: Diacylglycerol
FAE: Fatty acid elongation
LAG: Genes encoding for longevity-assurance
G3P: Glycerol-3-phosphate
GPI: Glycosylphosphatidylinositol
IPC: Inositol phosphoryl ceramide
ILVs: Intraluminal vesicles
LPG: Lipophosphoglycanes
LPPG: Lipophosphopeptideglycanes
LDL: Low density lipoprotein
LBPA: Lysobisphosphatidic acid
LPA: Lysophosphatidic acid
LPL: Lysophospholipids
MVBs: Multivesicular bodies
NPC1/NPC2: Niemann-Pick type C1 and C2 proteins
PanK: Pantothenate kinase
PA: Phosphatidic acid
PtdIspn: Phosphatidyl isopropanolamine phospholipid
PC: Phosphatidylcholine
PE: Phosphatidylethanolamine
PG: Phosphatidylglycerol
PIs: Phosphatidylinositides
PI: Phosphatidylinositol
PI(4,5)P_2_, PI(3,5)P_2_, PI(3,4)P_2_: Phosphatidylinositol bi-phosphate
PI-3P, PI-4P, and PI-5P: Phosphatidylinositol monophosphate
PIPK: Phosphatidylinositol phosphate kinase
PI(3,4,5)P_3_: Phosphatidylinositol (3,4,5) tri-phosphate
PS: Phosphatidylserine
PLC: Phospholipase C
PGE2: Prostaglandin E2
SPT: Serine palmitoyl transferase
SL: Sphingolipids
SM: Sphingomyelin
SMA: Sphingomyelinases
TA: Transacylases
EhVPSs: Vacuolar protein sorting proteins.
